# Natural history of dental caries in very young Australian children

**DOI:** 10.1111/ipd.12169

**Published:** 2015-05-13

**Authors:** Mark Gussy, Rosie Ashbolt, Lauren Carpenter, Monica Virgo‐Milton, Hanny Calache, Stuart Dashper, Pamela Leong, Andrea de Silva, Alysha de Livera, Julie Simpson, Elizabeth Waters

**Affiliations:** ^1^ Department of Dentistry and Oral HealthLa Trobe Rural Health SchoolLa Trobe UniversityBendigoVic.Australia; ^2^ Melbourne School of Population & Global HealthThe University of MelbourneMelbourneVic.Australia; ^3^ Jack Brockhoff Child Health & Wellbeing ProgramMelbourne School of Population & Global HealthThe University of MelbourneCarltonVic.Australia; ^4^ Dental Health Services VictoriaCarltonVic.Australia; ^5^ Melbourne Dental SchoolUniversity of MelbourneCarltonVic.Australia; ^6^ Department of Dentistry and Oral HealthLa Trobe UniversityMelbourneVic.Australia; ^7^ Oral Health Cooperative Research CentreMelbourne Dental SchoolThe University of MelbourneCarltonVic.Australia; ^8^ Early Life Epigenetics GroupMurdoch Childrens Research InstituteRoyal Childrens HospitalParkvilleVic.Australia; ^9^ Centre for Applied Oral Health ResearchDental Health Services VictoriaCarltonVic.Australia; ^10^ Centre for Epidemiology and BiostatisticsMelbourne School of Population & Global HealthThe University of MelbourneCarltonVic.Australia

## Abstract

**Background:**

Whilst the global burden of caries is increasing, the trajectory of decay in young children and the point at which prevention should occur has not been well established.

**Aim:**

To identify the ‘natural history’ of dental caries in early childhood.

**Design:**

A birth cohort study was established with 467 mother/child dyads followed at 1, 6, 12, 18, and 36 months of age. Parent‐completed surveys captured demographic, social, and behavioural data, and oral examinations provided clinical and data.

**Results:**

Eight per cent of children (95% confidence interval (CI): 5–12%) at 18 months and 23% (95% CI: 18–28%) at 36 months experienced decay. Interesting lesion behaviour was found between 18 and 36 months, with rapid development of new lesions on sound teeth (70% of teeth, 95% CI: 63–76%) and regression of many lesions from non‐cavitated lesions to sound (23% of teeth, 95% CI: 17–30%). Significant associations were found between soft drink consumption and lesion progression.

**Conclusions:**

Findings suggest optimal time periods for screening and prevention of a disease which significantly impacts multiple health and well‐being outcomes across the life course.

## Introduction

The disease of early childhood caries (ECC) is defined as the presence of one or more decayed (non‐cavitated or cavitated lesions), missing (due to caries), or filled tooth surfaces in any primary tooth in a child under the age of 6 years^1^. In children younger than 3 years of age, any sign of smooth‐surface caries is indicative of severe early childhood caries^1^. If left to progress, the short‐term sequelae include pain, infection, delayed growth, and restriction of normal daily activities^2^. In the longer term, ECC is the best predictor of future dental caries^3^. Internationally, the reported prevalence of ECC ranges from 12% to 70% depending on the population^4^, ^5^, and the disease process is initiated much earlier than school entry age, with 28% of US two‐ to five‐year‐olds having visually evident caries^6^. In Australia, approximately 50% of children enter primary school with largely untreated, frank carious cavitations^7^, suggesting that opportunities have been missed to arrest the disease, which is possible up to the point of surface breakdown or cavitation.

There is emerging evidence that different teeth and/or tooth surfaces may be more susceptible to disease progression and early detection, and assessment of carious lesions can help predict which lesions are most likely to progress to cavitation^8^. These could be targeted for evidence‐based therapeutic and behavioural interventions. Understanding the time points when there is greatest risk and maximum opportunity for prevention, and aligning these times with the health and clinical care settings and services which children and their parents engage with, might impact on disease trajectory.

Few epidemiological studies have been conducted with children under the age of 5 years, and these are largely cross‐sectional^9^. The most recent Australian study reported a mean value of 1.94 teeth with caries in four‐year‐old children^7^, with the mean score of groups, where a large proportion of the sample had no disease (60% were caries free recording a dmft of 0), masking the true pattern and burden of disease. The SiC^10^ score 10 for four‐year‐olds was five times higher (9.97 teeth affected) than the mean dmft for the whole sample^7^. In this study, cavitation was used as the threshold of disease meaning the true disease levels are likely to be greater.

Given the importance of early lesions, it is surprising that many researchers continue to use cavitation as a primary dental variable. The main argument supporting this approach is that detection of pre‐cavitated lesions is difficult, unreliable and/or time‐consuming/resource consuming^11^. Minimal intervention dentistry principles articulated by the Federation Dentaire Internationale state that cavitation should not be considered the threshold for disease detection but rather as a failure of prevention or therapeutic control^12^.

Prospective studies of dental caries progression require the use of measures that quantify the carious lesion, for instance the clinical manifestation of the disease process at different stages over time, up to the point of cavitation.

In 2008, we commenced a birth cohort study called VicGeneration (VicGen) to determine (a) the pattern of dental caries as teeth enter the mouth and (b) the prevalence of dental caries in children at four age points between zero and 3 years^13^.

The aim of this study, the first of its kind, was to report on the natural history of dental caries in young children aged between zero and 3 years and to assess lesion behaviour against a number of known risk and protective factors, that is plaque control, sweetened beverage consumption, and fluoride exposure.

## Material and methods

### Participants

Participants were recruited from maternal and child health (MCH) centres in six local government areas (metropolitan, regional, and rural) in Victoria, Australia. New mothers (around one month post‐natal) and their infants attending child health and well‐being checks were given an information pack and invited by MCH nurses to participate. Criteria for exclusion were families who intended to move location within the next 12 months; children with special healthcare needs; presence of severe illness in the family; or the presence of parental mental illness. Contact details of 917 mothers who indicated interest were forwarded to researchers, who contacted them via phone. Forty‐four mothers were unable to be contacted despite multiple attempts, and of the remaining 873 contacted, 467 agreed to participate.

### Study visits

Data were collected during five visits at time points 1, 6, 12, 18, and 36 months of age. The majority of visits were conducted in the participant's home, with a small proportion conducted at the local MCH or Community Health Centre. Visits were conducted by one research assistant and one dentist or oral health therapist. This paper describes the results from the caries examination for the primary teeth of children at the six‐month (study visit two: SV2), 12‐month (study visit three: SV3), 18‐month (study visit four: SV4), and 36‐month (study visit five: SV5) time points. Study visit one (SV1) data were not included in the analysis as all infants were pre‐dentate. Participants reported their child's brushing behaviours and beverage consumption in the questionnaire administered at study visit four. Independent data on community water fluoridation (by postcode) were used to determine fluoridation status.

### Oral assessment

The International Caries Detection and Assessment System (ICDAS II) was used, which has been developed for clinical use in caries management, and for epidemiology, as the measure correlates the clinical appearance of a lesion with its histological depth or severity^14^. This measure provides triggers for clinical care and for predicting which lesions will progress to cavitation and speed of progression^8^. Research on the ICDAS II shows it is valid for use within children, comparable to standard criteria in epidemiological surveys^15^, and has acceptable validity and reproducibility for all codes when used with primary molar teeth^16^.

The need to collect data in the field rather than a dental clinic setting precluded the use of compressed air; therefore, Code 1 lesions could not be recorded unless they were located in pits or fissures and rated as a change in colour ‘due to caries which is not consistent with the clinical appearance of sound enamel and is limited to the confines of the pit and fissure area’^17^. All detectable Code 1 lesions were recorded as ICDAS II Code 2.

Children were examined in the ‘lap to lap’ position with the examiner seated behind the child's head and the child lying on their mother's lap. Mothers were examined sitting in a chair with the examiner behind them. Lighting was in the form of a head‐mounted lamp. No radiographs were exposed. Where plaque/debris prevented visualisation of tooth surface, it was removed with gauze.

### Examiner training

All examiners were trained to conduct oral examinations using the ICDAS II method. A qualified paediatric dentist led examiners through the ICDAS II e‐learning package^18^. Calibration of examiners was performed after the training was completed using photographic slides of early to advanced lesions comparing the participant's results with those of the reference standard. Kappa scores were calculated for intra‐ and interexaminer reliability. The scores ranged from moderate to substantial agreement (intra‐examiner 0.55–0.83, interexaminer 0.52–0.71), which is comparable with other studies^19^, ^20^; examiners were recalibrated annually. Three examiners conducted the majority of examinations across the study period.

### Ethics approval

Ethics approval was approved by the University of Melbourne Human Research Ethics Committee (HREC 0722543 and HREC 1137124) and the Victorian Department of Education and Early Childhood Development. All participants provided informed written consent before baseline and again before SV5.

### Statistical analysis

ICDAS II measurements at each study visit were analysed using the statistical software package stata (StataCorp, College Station, TX, USA). These measurements were analysed by tracking decay per tooth per child over time at each ICDAS II level of caries and also grouped into non‐cavitated lesions (codes 1–2) and cavitated lesions (codes 3–6), including the SiC^10^ measure. Prevalence of children with dental decay and the proportion of teeth with lesions were calculated, with corresponding confidence intervals. Surface level data were used to determine the change in caries status between SV4 and SV5. Children were assigned to one of four categories: progression only, regression only, progression and regression, and no change. We used multinomial logistic regression to estimate the odds ratios of a number of risk factors and the association with the caries status (i.e., progression, regression, or no change). The risk factors examined were consumed fruit juice (yes, no), consumed soft drink (yes, no), living in a fluoridated area at baseline (yes, no), and brushing with fluoridated toothpaste (yes, no). The regression only category was used as the reference category to allow for comparison between this and the progression only group. Only four children experienced both progression and regression so we did not include this group in our analysis.

## Results

### Cohort

The number of infants recruited into the study at baseline was 467: 48% (*n* = 227) were female. The majority (74%) of mothers spoke English at home, 65% were born in Australia, and 38% had a healthcare card (Table 1). India and Vietnam were the dominant maternal countries of birth outside of Australia. Sixty five per cent (65%) of parents resided in urban or outer urban Melbourne, and 35% in country or regional areas. The majority of participants (76%) lived in fluoridated areas.

**Table 1:** Demographic, socio‐economic, environmental, and educational profiles of participants at each study visit.^a^

	SV1	SV2	SV3	SV4	SV5
	*n* = 467^a^	*n* = 378^a^	*n* = 333^a^	*n* = 376^a^	*n* = 269^a^
Child age^b^ (months)	1·9 (0·9)	7·8 (1·4)	13·4 (2·1)	20·1 (2·4)	40·0 (3·9)
Mother age at baseline^b^ (years)	30·2 (5·3)	30·5 (5·3)	30·5 (5·2)	30·8 (5·1)	31·3 (5·1)
Child gender male^c^	240 [51]	198 [52]	178 [53]	200 [53]	140 [52]
Had healthcare card^c^	178 [38]	132 [35]	106 [32]	125 [33]	73 [27]
Mother born in Australia^c^	305 [65]	260 [69]	241 [72]	261 [69]	193 [72]
Mother speak English at home^c^	344 [74]	296 [78]	269 [81]	293 [78]	216 [80]
Mother education level^c^					
No post‐school education	98 [21]	75 [20]	58 [17]	69 [18]	36 [13]
Certificate/diploma/advanced diploma/apprenticeship	186 [40]	152 [40]	133 [40]	151 [40]	103 [38]
Bachelor degree or higher	180 [39]	150 [40]	142 [43]	156 [41]	129 [48]
Residential Region^c^					
Metropolitan	303 [65]	240 [63]	198 [59]	234 [62]	160 [59]
Regional	53 [11]	47 [12]	47 [14]	49 [13]	43 [16]
Rural	111 [24]	91 [24]	88 [26]	94 [25]	66 [25]
Water fluoridation^c^					
Live in fluoridated neighbourhood	354 [76]	286 [76]	245 [74]	281 [75]	200 [74]
Live in non‐fluoridated neighbourhood	113 [24]	92 [24]	88 [26]	96 [25]	69 [26]

a All participants, including a few children whose data were excluded from the subsequent statistical analysis due to invalid odontograms (SV2 *n* = 4; SV3 *n* = 3; SV5 *n* = 1). Baseline information for 15 participants was not available.

b Values are mean (standard deviation).

c Values are number [%].

At no time were any teeth recorded as missing due to caries, or filled; therefore, the dmft comprises only the decayed (cavitated and non‐cavitated) component. At SV1, where the mean age of infants was 1.9 months, no teeth were recorded. Table 2 summarises dental caries in primary dentition for all ICDAS classifications at each study visit. The median number of teeth per child gradually increased from SV2 to SV5 with progressively narrowing ranges. All lesions reported, where the mean children ages were between 7.8 and 20.1 months, were non‐cavitated lesions. The first cavitated lesions were detected at SV5, when mean child age was 40 months.

**Table 2:** Summary of dental caries in primary dentition for all ICDAS classifications at each study visit, showing number of teeth (number of children with teeth)* and median number of teeth per child [range]^†^

	Total	d1	d2	d3	d4	d5	d6	Non‐cavitated	Cavitated	Any ECC
SV2 (*N* = 374)	508 (185)* 0 [0,8]^†^	1 (1)*	3 (2)*	0 (0)*	0 (0)*	0 (0)*	0 (0)*	4 (3)*	0 (0)*	4 (3)*
SV3 (*N* = 330)	2228 (325)* 6.5 [0,16]^†^	5 (3)*	5 (2)*	0 (0)*	0 (0)*	0 (0)*	0 (0)*	10 (5)*	0 (0)*	10 (5)*
SV4 (*N* = 390)	5446 (390)* 16 [4,20]^†^	38 (17)*	40 (13)*	0 (0)*	0 (0)*	0 (0)*	0 (0)*	78 (27)*	0 (0)*	78 (27)*
SV5 (*N* = 270)	5387 (270)* 20 [16,20]^†^	31 (17)*	100 (43)*	20 (9)*	2 (2)*	13 (8)*	2 (2)*	131 (55)*	37 (15)*	168 (62)*

**Figure 1:**
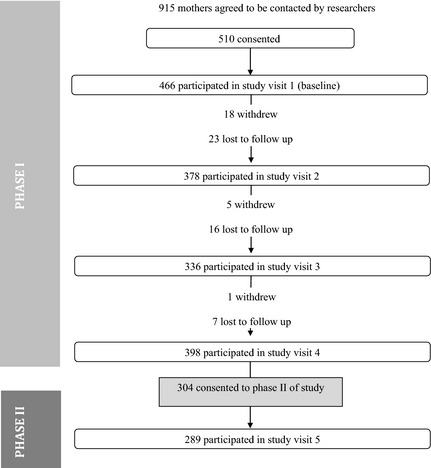
Flow of participants through the study.

The speed of caries acquisition and progression of decay can be established by examining caries status per tooth per child at SV4 and SV5. At SV5, the number of children who participated in the study was 270. Figure 1 shows the flow of participants through the VicGen study between baseline and SV5. The figure depicts the number of participants who completed at least one of the three aspects of data collection (saliva collection, oral assessment, and questionnaire) at each wave.

Of these, 268 children also participated at SV4, allowing us to track dental decay per child per tooth during the 18‐month time period between SV4 and SV5. At SV4, 8% (95% CI: 5–12%) of these children experienced dental decay which progressed to 23% (95% CI: 18–28%) at SV5 (Table 3). The median number and range of teeth with any caries experience for the 10% of children who recorded the highest number of lesions (SiC^10^) was 1 [0, 11] at SV4 increasing to 4 [2, 16] at SV5. For these same children, the range of teeth with cavitated (codes 3–6) lesions increased from [0, 0] at SV4 to [0, 5] at SV5.

**Table 3:** Median number and range of teeth with caries experience, SiC^10^ scores, and prevalence of caries at various ICDAS thresholds, at SV4 and SV5

Decay status	SV4				SV5			
	All children		Sic10		All children		Sic10	
	Median [range]^a^	*n* (%)^b^	Median [range]^a^	*n* (%)^b^	Median [range]^a^	*n* (%)^b^	Median [range]^a^	*n* (%)^b^
Non‐cavitated	0 [0,11]	21 (7.8)	0 [0,11]	21 (7.8)	0 [0,11]	54 (20.1)	3 [2,11]	26 (9.7)
Cavitated	0 [0,0]	0 (0)	0 [0,0]	0 (0)	0 [0,5]	15 (5.6)	1 [0,5]	15 (5.6)
Any caries	0 [0,11]	21 (7.8)	1 [0,11]	21 (7.8)	0 [0,16]	61 (22.8)	4 [2,16]	26 (9.7)

a Median number [range] of teeth with caries experience.

b Number of children (percentage) with caries experience.

During the 18‐month period between SV4 and SV5 where interesting lesion behaviour was observed, complete case analysis was used to manage the missing values. At either SV4 or SV5, 191 teeth from 73 children were recorded as having any caries experience (Table 4). Of the 191 teeth, 70% (95% CI: 63–76%) had lesions that had manifested on previously sound teeth and 23% (95% CI: 17–30%) regressed from decay to sound at some point between these two examination periods (that is between mean ages of 20 and 40 months). No implausible regressions were recorded (i.e., from cavitated lesion to a non‐cavitated lesion or sound tooth). Further, at SV5, the majority of ECC occurred in the posterior teeth, and these lesions were mostly non‐cavitated lesions.

**Table 4:** Decay status and progression of teeth with any caries experience at SV4 and SV5

Tooth	Study Visit						Progression					
	Study visit 4			Study visit 5			Decay to sound		Sound to decay		Non‐caviation to cavitation	Non‐caviation to non‐cavitation
	Sound	Uncavitated lesions	Cavitated lesions	Sound	Uncavitated lesions	Cavitated lesions	Non‐cavitation to sound	Cavitation to sound	Sound to non‐cavitation	Sound to cavitation		
55	13	0	0	0	11	2	0	0	11	2	0	0
54	5	7	0	6	6	0	6	0	5	0	0	1
53	3	2	0	2	2	1	2	0	2	1	0	0
52	5	3	0	1	4	3	1	0	4	1	2	0
51	5	6	0	4	2	5	4	0	2	3	2	0
61	4	7	0	4	4	3	4	0	3	1	2	1
62	4	3	0	0	4	3	0	0	3	1	2	1
63	4	2	0	2	4	0	2	0	4	0	0	0
64	5	5	0	4	5	1	4	0	4	1	0	1
65	16	0	0	0	12	4	0	0	12	4	0	0
85	17	0	0	0	14	3	0	0	14	3	0	0
84	13	5	0	4	10	4	4	0	9	4	0	1
83	4	1	0	1	3	1	1	0	3	1	0	0
82	1	1	0	1	1	0	1	0	1	0	0	0
81	1	3	0	3	1	0	3	0	1	0	0	0
71	0	4	0	4	0	0	4	0	0	0	0	0
72	0	2	0	2	0	0	2	0	0	0	0	0
73	1	1	0	1	1	0	1	0	1	0	0	0
74	10	6	0	5	9	2	5	0	8	2	0	1
75	22	0	0	0	20	2	0	0	20	2	0	0
Total number of teeth (%)	133 (69.6)	58 (30.4)	0 (0)	44 (23.0)	113 (59.1)	34 (17.8)	44 (23)	0 (0)	107 (56.0)	26 (13.6)	8 (4.2)	6 (3.1)
Number of teeth per child	2.4	2.8	–	2.3	2.2	2.4	2.3	–	2.1	2.2	2.7	1.5

When comparing lesion progression to no change, those with reported consumption of soft drinks had significantly increased odds (314%) of lesion progression. No statistically significant associations were shown between lesion progression or regression and consumption of fruit juice, exposure to water fluoridation, or brushing with fluoridated paste more than once a day (Table 5).

**Table 5:** Unadjusted multinomial logistic regression and change in tooth status between study visits 4 and 5

	No change (*n* = 214^a^)		Progression only (*n* = 23^a^)			Regression only (*n* = 16^b^)		
	Yes (%)	OR (95% CI)	Yes (%)	OR (95% CI)	*P*‐value	Yes (%)	OR (95% CI)	*P*‐value
Drinks fruit juice	56.1	1	69.6	1.79 (0.71, 4.53)	0.22	43.8	0.61 (0.22, 1.70)	0.34
Drinks soft drink	15	1	43.5	4.12 (1.71, 10.42)	0.002	18.8	1.27 (0.34, 4.69)	0.72
Did not live in fluoridated area at baseline	22.4	1	34.8	1.84 (0.74, 4.61)	0.19	15.4	0.63 (0.13, 2.93)	0.56
Did not brush with fluoridated paste at least once per day	87.1	1	86.4	0.94 (0.26, 3.40)	0.93	75.0	0.45 (0.13, 1.49)	0.19

Abbreviations: OR = odds ratio; 95% CI = 95% confidence interval.

Total *N* = 253; reference category for regression: no change.

Children with progression and regression in their mouths excluded (*n* = 4).

a Brushing behaviour; progression only group *n* = 22; no change group: *n* = 201.

b Fluoride variable *n* = 13.

## Discussion

The novel features of the VicGen study are that it is prospective in design, with the cohort commencing at a very young age (2 months) and being assessed five times until the age of approximately 36 months. This has enabled a unique prospective examination of the incidence and progression of dental caries as teeth enter the mouth over a period of significant developmental change. By comparison with the existing research, other studies in Australia^21^, ^22^ and internationally^23^, ^24^ are largely cross‐sectional or, if prospective, involve older children^8^. Contextual factors in developing countries reduce ability to compare the caries experience of these children with other published work^25^.

The prevalence of cavitated teeth (codes 3–6) for children at SV5 with a mean age of 40 months was around two per cent, and when considering all lesions (codes 1–6), the caries prevalence was 20%. The only comparably aged sample resided in non‐fluoridated rural communities where prevalence of pre‐cavitated and cavitated teeth was 20%^26^ and 29%, respectively. The sample in our study was more likely to live in metropolitan Melbourne and, regardless of location of residence, more likely to live in a fluoridated area.

Early childhood caries has the distinct pattern of affecting first the anterior maxillary teeth, then the fissures of the molars, and finally the proximal surfaces of the molars with the lower anterior teeth least affected^27^. Our data as expected show more advanced lesions in anterior maxillary teeth when the children are older and more new non‐cavitated lesions associated with the molars.

The rapid development of new lesions on previously sound teeth in the 18‐month period between SV4 and SV5 is of particular interest as is the regression of lesions from non‐cavitated lesions to sound. This suggests this is a dynamic period with new disease development and for prevention, slowing, or reversal of disease. It may be that this is an important period, with the introduction of both new risk factors (such as foods and drinks), and changes in plaque biofilm, as well as protective factors (such as tooth cleaning with fluoridated toothpaste and exposure to fluoridated water), occurring in parallel. The balance of these risk and protective factors may be important in setting the trajectory for lifelong oral health.

Given our observations and those of the few other prospective Australian studies, together with the reported cross‐sectional survey data for four‐ and five‐year‐olds^7^, it appears that the period between 18 months and 40 months of age may be a significant period for the development of advanced non‐cavitated lesions (ICDAS II Code 2) and that the following 18 months could represent a period where the majority of cavitated lesions seen at school entry age manifest. When associations with the well‐established risk and protective factors (diet, plaque control, and fluoride exposure) were tested, a significant relationship with lesion progression was shown for soft drink consumption but not fruit juice consumption, brushing twice daily with a fluoride toothpaste nor with exposure to community water fluoridation. Previous studies have highlighted the potential for sweet beverages to increase risk of dental caries^28^, ^29^. Our finding of significant associations between soft drinks and progression of lesions but not fruit juice supports recent findings from case–control studies^30^. Like Sohn *et al*. we speculate that carbonated soft drinks likely have greater cariogenic potential because they are highly acidic and have added buffering agents which maintain low pH. These results make an important contribution to the literature, because there are no other studies known prospective studies that examine this phenomenon in children of this age.

A limitation of this study is the approach taken to examiner calibration. The nature of the field examinations and the age of the included children required a pragmatic approach to training and assessment of examiner reliability. Examination of children aged 6–48 months is challenging, and the burden for such young children and parents of multiple examinations by several examiners was not considered appropriate. In addition, the high number of 00 codes would have necessitated a large number to be screened in preparation for the reliability testing or a large number to be subjected to the reliability testing process to capture sufficient numbers of each of the caries codes. Additionally, the lack of compressed air in the field limited our ability to measure early non‐cavitated lesions (ICDAS II Code 1) on smooth surfaces. This may have resulted in under‐recording of early non‐cavitated lesions that are histologically less advanced and therefore simpler to arrest or reverse. Finally, the loss of participants by SV5 has reduced the power of the study and we caution readers to bear this in mind when considering the study results. The mothers of children included in SV5 were older and more highly educated than the average enrolled at the beginning of the study.

The VicGen study is one of few internationally that have tracked the natural history of dental caries in children from birth. Very little carious activity was detected in the first 18 months of life. In the following 18 months, however, there was a steep increase in non‐cavitated lesions, particularly in the molar teeth, and the appearance of the first cavitated lesions. Additionally, this was a period when many non‐cavitated lesions were inactive (i.e., did not progress to cavity) or regressed. The consumption of carbonated soft drinks significantly increased the risk of lesion progression. Study findings add to the understanding of the relative contribution of the multiple risk and protective factors for ECC at this stage of childhood and suggest that soft drink use in young children should be avoided to modify the disease trajectory and promote lifelong oral health.

**Why this paper is important to paediatric dentists**

- Understanding progression of decay as teeth enter the mouth is essential for effective prevention and clinical care.
- The trajectory of dental decay in young children, the influence of risk and protective factors on early childhood caries, and the age and which prevention should commence has not been well established.
- Internationally, we are witnessing an avoidable increase in dental caries. This study is one of few that have tracked the natural history of dental caries in children from birth, and the findings add to the understanding of the relative contribution of the multiple risk and protective factors for ECC at this stage of childhood.

## Conflict of interest

Dr. de Silva reports grants from National Health and Medical Research Council, grants from Foundation for Children, grants and personal fees from Dental Health Services Victoria, non‐financial support from Victorian Government, Department of Education and Early Childhood Development, during the conduct of the study. Grants and Non‐financial support from Colgate‐Palmolive Pty Ltd (NSW), grants and non‐financial support from Victorian Department of Health, grants from Australian Research Council, personal fees and non‐financial support from University of Melbourne, outside the submitted work. Dr. Dashper reports grants from National Health and Medical Research Council, during the conduct of the study. Ms. Carpenter reports grants from National Health and Medical Research Council, grants from Financial Markets Foundation for Children, grants and non‐financial support from Dental Health Services Victoria, other from Victorian Government, Department of Education and Early Childhood Development, during the conduct of the study. Other from Colgate Australia, outside the submitted work. Ms. Virgo‐Milton reports grants from National Health and Medical Research Council, grants from Financial Markets Foundation for Children, grants from Dental Health Services Victoria during the conduct of the study. The rest of the authors declare no conflict of interests.

## Author contributions

Mark Gussy participated in project design and management, planning of data collection processes, data interpretation, and writing of the manuscript. Rosie Ashbolt participated in data management, statistical analysis, interpretation, and writing of the manuscript. Hanny Calache participated in study design, examiner training and calibration, data interpretation, and writing of the manuscript. Lauren Carpenter participated in data collection, management, analysis, and interpretation. Monica Virgo Milton participated in data collection, management, and interpretation. Pamela Leong participated in data collection, examiner training and calibration, and writing of the manuscript. Stuart Dashper participated in project design and management, data interpretation, and writing of the manuscript. Elizabeth Waters participated in project design and management, data collection, data management and analysis, data interpretation, and writing of the manuscript. Andrea de Silva participated in project management and oversight, data collection methods and procedures, data management, data interpretation, and writing of the manuscript. Alysha De Livera participated in statistical analysis, interpretation, and writing of the manuscript. Julie Simpson participated in statistical analysis, interpretation, and writing of the manuscript.
